# Explorando la intersección entre cambio climático, género y seguridad alimentaria en Latinoamérica

**DOI:** 10.7705/biomedica.7901

**Published:** 2025-11-27

**Authors:** Natalia Cediel-Becerra, Diana Sánchez-Arévalo

**Affiliations:** 1 Facultad de Ciencias Agropecuarias, Universidad de La Salle, Bogotá, D. C., Colombia Universidad de la Salle Facultad de Ciencias Agropecuarias Universidad de La Salle Bogotá, D. C. Colombia; 2 Facultad de Ciencias Agropecuarias, Universidad de La Amazonía, Florencia, Caquetá, Colombia Universidad de la Amazonia Facultad de Ciencias Agropecuarias Universidad de La Amazonía Florencia Caquetá Colombia

**Keywords:** abastecimiento de alimentos, cambio climático, identidad de género, equidad, adaptación, América Latina., Food supply, climate change, gender identity, equity, adaptation, Latin America

## Abstract

**Introducción.:**

Las consecuencias del cambio climático en las mujeres de los países de Latinoamérica son notablemente más serias a causa de las brechas persistentes en la educación y en el acceso a la tierra y a los servicios de información. Estas desigualdades se profundizan e incrementan los riesgos sanitarios y el deterioro del bienestar y amenazan la subsistencia de los grupos femeninos rurales.

**Objetivo.:**

Describir la relación entre el cambio climático y la seguridad alimentaria con enfoque de género en Latinoamérica.

**Materiales y métodos.:**

Se llevó a cabo una revisión exploratoria en las bases de datos Redalyc, SciELO, Google Scholar, EBSCO, Web of Science y Scopus. Se analizaron 36 documentos publicados entre el 2010 y el 2022, con énfasis en los países de Latinoamérica.

**Resultados.:**

Los eventos climáticos más frecuentes fueron: sequías, inundaciones, aumento de la temperatura y deslizamientos, los cuales generaron limitaciones en el abastecimiento de alimentos. Se identificaron brechas en salud, acceso a los recursos e información, seguridad y derechos humanos, que reproducen vulnerabilidades sociales y dificultan la efectividad de las políticas públicas destinadas a mitigar el impacto del cambio climático y las secuelas sociales de la pandemia. Los riesgos asociados con el cambio climático son particularmente graves para las mujeres y niñas indígenas y afrodescendientes, las mujeres mayores, las personas de la comunidad LGBTIQ+, las mujeres con discapacidad, las mujeres en situación de migración y aquellas que residen en zonas rurales, remotas o expuestas a desastres y conflictos.

**Conclusiones.:**

El cambio climático no es neutral al género y persiste una brecha en la implementación de las políticas de adaptación con enfoque de género.

## Abstract (en)

**Introduction.:**

The consequences of climate change for women in Latin American countries are more severe due to persistent gaps in education, land ownership and access to information services. These inequities heighten health, welfare and livelihood risks among rural women.

**Objective.:**

To describe the relationship between climate change and food security from a gender perspective in Latin America.

**Materials and methods.:**

An exploratory review was conducted in Redalyc, SciELO, Google Scholar, EBSCO, Web of Science and Scopus. We analyzed 36 documents published between 2010 and 2022 focusing on Latin American countries

**Results.:**

The most frequently described extreme events were droughts, floods, rising temperatures, and landslides, all of which contributed to food supply shortages. Evidence shows persistent gaps in health, access to resources and information, security and human rights, which perpetuate social vulnerability and hinder the effectiveness of public policies addressing the impacts of climate change and the social consequences of the pandemic. Climate-related risks are particularly severe for indigenous and Afro-descendant women and girls, older women, LGBTIQ+ people, women with disabilities, women in migration contexts, and those living in rural, remote or disaster- and conflict-prone areas.

**Conclusions.:**

Climate change is not gender-neutral, and there remains a gap in the implementation of gender-sensitive climate adaptation policies.

El cambio climático se refiere a los cambios en el estado del clima que pueden identificarse por variaciones en la temperatura promedio o en la variabilidad de sus propiedades y que persisten durante períodos prolongados, generalmente décadas o más ^1^^,^^2^. Las principales emisiones de gases de efecto invernadero que provocan el cambio climático corresponden a las de dióxido de carbono, óxido nitroso y metano. Estos gases son generados por actividades humanas como la tala de árboles, la quema de combustibles fósiles, los cambios en el uso del suelo, la ganadería, el uso de fertilizantes químicos y diversos procesos industriales ^2^. El cambio climático antropogénico ha provocado cambios generalizados en la atmósfera, el océano, la criósfera y la biósfera, lo cual ha derivado en efectos adversos, pérdidas y daños relacionados con la naturaleza y las actividades humanas ^3^.

Entre los efectos más negativos del cambio climático sobre las necesidades humanas básicas, se encuentran la escasez de agua y la falta de alimentos, con consecuencias significativas para la salud de millones de personas ^4^. Se ha estimado que, sin medidas para mitigar el cambio climático, la mitad de la población mundial podría vivir en países con estrés hídrico hacia el 2050, de los cuales el 80 % correspondería a países en desarrollo. Los grupos y personas excluidas -por factores sociales, económicos, culturales, políticos o institucionales- son más vulnerables a estos impactos, así como ante las medidas de adaptación o mitigación que deban implementarse para enfrentar sus consecuencias ^5^.

Según la *Food and Agriculture Organization of the United Nations* (FAO), la seguridad alimentaria existe cuando todas las personas tienen, en todo momento, acceso físico, social y económico a alimentos suficientes, inocuos y nutritivos que satisfagan sus necesidades energéticas diarias y preferencias alimentarias para llevar una vida activa y sana ^(6)^. Sus cuatro dimensiones incluyen:

- la disponibilidad, entendida como la posibilidad de contar con alimentos en cualquier momento;
- el acceso, relacionado con la provisión de alimentos a escala nacional, regional o local;
- el aprovechamiento biológico y de calidad, es decir, la capacidad para utilizar los alimentos, y
- la estabilidad, que se refiere al acceso permanente de una población a alimentos adecuados.

Según la FAO, los conflictos, la variabilidad del clima y los fenómenos climáticos extremos, la falta de acceso a dietas saludables, los entornos alimentarios poco saludables y los altos índices de desigualdad social siguen generando inseguridad alimentaria y malnutrición en todo el mundo. En particular, la variabilidad climática se presenta cada vez con mayor frecuencia e intensidad, lo que afecta de forma negativa la disponibilidad y el acceso físico y económico a los alimentos por parte de la población, así como su uso y estabilidad, la cual está fuertemente influenciada por el aumento de las temperaturas tanto en las zonas rurales como en las urbanas ^6^.

Cabe destacar que el concepto de seguridad alimentaria ha evolucionado durante las últimas décadas y actualmente se reconoce también la importancia fundamental del derecho a la alimentación. Mientras la seguridad alimentaria se basa en necesidades que deben abordarse mediante políticas y programas, el derecho a la alimentación es de naturaleza jurídica, en el cual las personas son titulares de derechos y los Estados son titulares de obligaciones.

La variabilidad climática y los fenómenos extremos pueden afectar negativamente las cuatro dimensiones de la seguridad alimentaria de forma interrelacionada, lo que impacta de manera desigual a las personas y a sus medios de vida. Esta situación puede comprometer el derecho a una alimentación adecuada. Aunque la gobernanza de la seguridad alimentaria y la nutrición puede fortalecerse sin un reconocimiento explícito de este derecho en los marcos normativos de los países, su incorporación puede constituir una base esencial para garantizar el ejercicio de otros derechos fundamentales. El derecho a una alimentación adecuada se define como

«[...] el derecho a tener acceso, de manera regular, permanente y libre, sea directamente o mediante compra en dinero, a una alimentación cuantitativa y cualitativamente adecuada y suficiente, que corresponda a las tradiciones culturales de la población a la que pertenece el consumidor y garantice una vida física y mental, individual y colectiva, libre de angustias, satisfactoria y digna […]**»**^7^.

Por otro lado, el aumento de la temperatura y de la humedad provocado por el cambio climático favorece la transmisión de enfermedades infecciosas, mediante la proliferación y la diseminación de agentes patógenos en humanos y en animales, así como de plagas y especies invasoras. Estos factores afectan las condiciones de almacenamiento, transporte y conservación de los alimentos, lo cual incrementa el riesgo de enfermedades de transmisión alimentaria ^3^^,^^8^. Estos eventos causan disminución de los ingresos e impactan sobre todo a poblaciones vulnerables dedicadas a las actividades agrícolas. Es importante destacar que las desigualdades estructurales -económicas, políticas y sociales- incrementan la vulnerabilidad de las mujeres rurales frente a los fenómenos climáticos extremos, ya que constituyen una carga adicional que puede empujarlas a la pobreza al afectar gravemente su acceso a diversos medios de vida ^7^.

Latinoamérica se caracteriza por ser la región más desigual del mundo, a pesar de no ser la más pobre. Presenta el coeficiente de Gini más alto a nivel global: el 10 % más rico concentra entre el 30 y el 40 % de los ingresos totales, mientras que el 40 % más pobre accede solo al 10 o 15 %. Las áreas rurales concentran los mayores índices de pobreza y malnutrición ^9^. Según el “Panorama regional de la seguridad alimentaria y la nutrición” ^7^, se estima que, en el 2023, la prevalencia de inseguridad alimentaria moderada o grave en la región fue del 28,2 %, lo cual afectó a cerca de 186,7 millones de personas, con una prevalencia del 32,2 % en las zonas rurales frente a otra de 26 % en las zonas urbanas. En la región de Latinoamérica y el Caribe, se presenta la mayor brecha entre hombres y mujeres en este indicador, alcanzando 5,2 puntos porcentuales ^7^. Particularmente, los pueblos originarios aborígenes y las mujeres suelen sufrir las consecuencias de esta realidad ^10^. En su revisión, Ruderman y Núñez ^10^ constataron que la inseguridad alimentaria es un factor de riesgo para el exceso de peso en mujeres, especialmente en zonas rurales.

El impacto del cambio climático, los roles, las percepciones, la autonomía en la toma de decisiones y las medidas de adaptación, difieren según la pertenencia a determinados grupos sociales. ONU-Hábitat reporta que, tras analizar los desastres naturales de 141 países, se detectó que las mujeres y las niñas tienen 14 veces más probabilidades de morir que los hombres debido a las diferencias de género y a las desigualdades existentes relacionadas con sus derechos económicos y sociales ^11^.

Las mujeres y las niñas de grupos étnicos (minoritarios) o de áreas remotas y desfavorecidas pueden sufrir múltiples formas de exclusión y opresión. Cuando se producen desastres, se exacerban estas desigualdades, por lo que es más probable que se vean afectadas ^12^. La Organización de las Naciones Unidas (ONU) plantea que, al menos, cinco riesgos se exacerban en el contexto del cambio climático para las mujeres y las niñas: aumento de la violencia de género, favorecimiento del matrimonio infantil, incremento de la mortalidad neonatal, empeoramiento de otras condiciones de salud materno-infantil e interrupciones de los servicios de salud sexual y reproductiva ^11^. Estos hallazgos evidencian que la crisis climática constituye también una crisis de igualdad de género ^12^.

Actualmente, existe un vacío en la literatura publicada sobre la relación entre el cambio climático y la seguridad alimentaria en Latinoamérica desde un enfoque de género, lo que limita el conocimiento sobre las siguientes categorías de análisis: vulnerabilidad, roles de género, medidas de adaptación y recursos o políticas para el empoderamiento de la mujer.

El objetivo de este estudio fue describir la relación entre el cambio climático y la seguridad alimentaria -en sus cuatro dimensiones- desde una perspectiva de género en Latinoamérica, considerando las categorías de análisis previamente mencionadas.

## Materiales y métodos

### Búsqueda y revisión bibliográfica

Se hizo una revisión de la literatura según la metodología descrita en el 2009 por Grant y Booth ^13^. Este tipo de exploración pretende buscar, evaluar y sintetizar sistemáticamente la evidencia disponible sobre un tópico de investigación, siguiendo las pautas establecidas para llevar a cabo una revisión.

### Pregunta de investigación, ecuación de búsqueda y bases de datos

El algoritmo de búsqueda se planteó a partir de la pregunta de investigación: ¿cuál es la relación existente entre el cambio climático y la seguridad alimentaria con una perspectiva de género en Latinoamérica, analizada mediante las categorías de vulnerabilidad, roles de hombres y mujeres, medidas para la adaptación al cambio climático y recursos para el empoderamiento de la mujer?

Entre abril y diciembre del 2022, se revisó la literatura publicada en los últimos doce años (2010-2022) que incluyó artículos de investigación original e informes de organizaciones internacionales especializadas. Se utilizaron las siguientes bases de datos: Redalyc, SciELO, Google Scholar, EBSCO, Web of Science, Scopus y PubMed, seleccionadas por su pertinencia frente a los escenarios latinoamericanos y sus procesos sociales y culturales. Se emplearon conjugaciones de palabras de búsqueda en español, inglés y portugués, definidas a partir de los descriptores DeCS y MeSH. Se realizaron variaciones en las fórmulas de búsqueda en función de mejorar su alcance en las diferentes bases de datos (tabla 1).

**Tabla 1:** Bases de datos y algoritmos de búsqueda utilizados en la revisión

Base de datos, fuente de información	Términos de búsqueda	Idioma	Número de artículos encontrados (aceptados)
Google scholar	“Adaptación, cambio climático, género, seguridad alimentaria, Latinoamérica”	Español	16.700 ^14^
Redalyc	“Cambio climático, género, seguridad alimentaria, Latinoamérica, vulnerabilidad”	Español	330 ^1^
Scielo	“Vulnerabilidad, cambio climático, género”	Español	7 ^2^
Google Scholar	“Seguranza alimentar, perspectiva de género, mudanza climática, adaptacao a desastres, Brasil”	Portugués	8.760 ^3^
Web of Science	“Gender, climate change, vulnerability, food security”	Inglés	14 ^2^
Scopus	“Climate change, vulnerability, gender”	Inglés	23 ^1^
EBSCO	“Gender, climate change, food security”	Inglés	72 ^2^
PUBMED	“Gender, climate change, vulnerability, food security”	Inglés	9 ^1^
Literatura gris	“Adaptación, cambio climático, género, seguridad alimentaria, Latinoamérica”	Español	10

### Selección de documentos relevantes

Para la inclusión de los documentos se utilizaron los siguientes criterios:

1. año de publicación (2010-2022),
2. documentos publicados en inglés, español o portugués,
3. estudios realizados en Latinoamérica o el Caribe, y
4. artículos que incluyeran o describieran alguna de las cuatro categorías de análisis con perspectiva de género referidas en la siguiente sección.

Los criterios de exclusión fueron:

1. artículos que abordaran el cambio climático sin mencionar aspectos relacionados con el género, o
2. artículos con enfoque de género que no estuvieran centrados en Latinoamérica.

Se revisaron los títulos y los resúmenes de los documentos recuperados y, en aquellos que cumplieron con los criterios de inclusión, se evalúo el texto completo.

### Extracción de datos y categorías de análisis

Luego de la revisión completa de cada artículo, se extrajeron los datos relevantes y se organizaron en una hoja de cálculo para su análisis. Las variables registradas incluyeron título, objetivos, métodos generales, población, país y texto correspondiente a las categorías de análisis: (i) vulnerabilidad y desigualdad de género relacionadas con el cambio climático; (ii) roles asumidos por hombres y mujeres, y percepción de dichos roles; (iii) medidas de adaptación al cambio climático, y (iv) políticas, planes gubernamentales y recursos para el empoderamiento de la mujer.

Se seleccionaron 90 artículos con base en el título y el resumen. Tras la lectura del texto completo, se excluyeron aquellos en los que no fue posible identificar las cuatro categorías de análisis propuestas. Se obtuvieron 26 artículos que cumplían con los criterios e idoneidad. De manera paralela, se hizo una búsqueda de la literatura gris (informes de organizaciones internacionales, documentos no publicados en revistas científicas y reportes institucionales), de la cual se seleccionaron 10 documentos que cumplían los criterios establecidos.

La información extraída de los 36 documentos se examinó en Mendeley, versión 1.69.1 (*software* de gestión de referencias desarrollado por Elsevier), mediante un análisis narrativo del contenido, como lo describieron Grant y Booth ^13^. Los documentos seleccionados se clasificaron según las cuatro dimensiones de seguridad alimentaria y las cuatro categorías de análisis.

## Resultados

**Figura 1:**
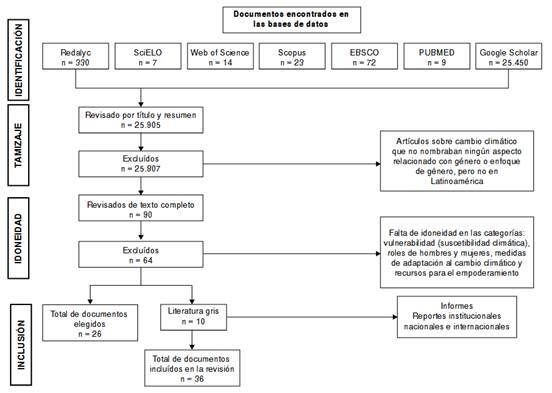
Diagrama de flujo del proceso de selección de los artículos incluidos en la revisión bibliográfica

El proceso de selección de los documentos incluidos en el análisis final se presenta en el diagrama de flujo PRISMA (figura 1) correspondiente a las búsquedas realizadas en las bases de datos consultadas. Para este artículo de revisión sistemática, se seleccionaron 36 documentos que cumplieron con los criterios de inclusión descritos en la metodología.

**Figura 2:**
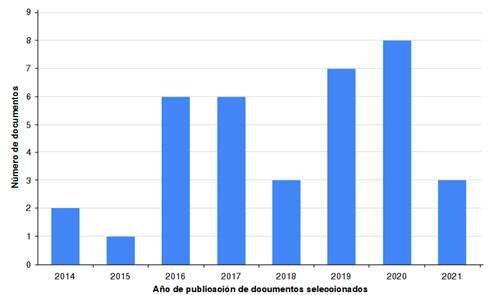
Distribución de los artículos seleccionados por año de publicación

La mayoría de los artículos identificados en Google Scholar y Redalyc fueron excluidos porque no incorporaban el enfoque de género o no describían el contexto de algún país de Latinoamérica. También, se incluyó literatura gris (informes institucionales) que cumplía los criterios necesarios para el análisis. Los 36 artículos e informes seleccionados se encuentran referenciados entre los números 14-49. Los países en los cuales se documentaron estudios sobre cambio climático, seguridad alimentaria y género en la región, fueron: México (n = 7), Honduras (n = 4), Brasil (n = 3), Guatemala (n = 3), Perú (n = 3), Cuba (n = 3), Ecuador (n = 3), Colombia (n = 2), Bolivia (n = 1), Chile (n = 1), Costa Rica (n = 1) y Nicaragua (n = 1). La distribución por año de publicación se muestra en la figura 2. Los resultados principales de las cuatro categorías de análisis se resumen en la tabla 2.

**Tabla 2:** Resultados del análisis de los artículos seleccionados según las cuatro categorías de análisis propuestas

Categoría	Referencias	Resultados principales
Vulnerabilidad de género frente al cambio climático
	^14^^-^^28^	Las mujeres y las niñas son los grupos más afectados alrededor del cambio climático. Al tener un limitado acceso a crédito, tierra, agua, capacitación y nuevas tecnologías, tienen menores ingresos, mayor dificultad para obtener trabajo remunerado y son más vulnerables a la violencia sexual. En situaciones de bajo acceso a servicios básicos, conflicto armado e inseguridad y explotación de los recursos naturales, se genera mayor vulnerabilidad económica, mayor presencia de efectos psicoemocionales, y pérdida de salud física y mental.
	^24^^,^^29^^-^^31^	La vulnerabilidad aumenta cuando se trata de mujeres indígenas, afrodescendientes y migrantes (ya sea por desastres naturales o por las privaciones de agua). Cuando la composición de los hogares es encabezada por mujeres y están ubicadas en zonas geográficamente más lejos de las ciudades, como ocurre en el nordeste de Brasil. Se genera vulnerabilidad cuando los esposos migran en busca de trabajo; por la alta tasa de familias constituidas por madres solteras y la dependencia de dichas mujeres de los recursos naturales locales para su sustento
Percepción de los roles en hombres y mujeres alrededor del cambio climático
	^14^^,^^17^^,^^19^^,^^20^^,^^23^^-^^26^^,^^29^^,^^32^^-^^38^	Las mujeres y niñas realizan las actividades de recolección de agua y leña en muchas comunidades rurales. El promedio por día es de 8-11 horas destinadas a actividades relacionadas con quehaceres del hogar. Comunidades indígenas de Guatemala evidenciaron reducción de la cantidad de agua disponible para cultivos y humedad en suelos, lo que generó mayor preocupación en las mujeres. Las funciones de hombre y mujer están establecidas en muchas comunidades (el hombre busca forraje, la mujer ordeña y realiza labores de hogar). Cuando las mujeres se dedican a la venta de productos, se crea una oportunidad de autonomía e independencia. En el nordeste brasilero, la contribución económica de las mujeres al presupuesto familiar no era valorada. En Panamá, el PAGCC tenía como meta la incorporación de la perspectiva de género en la Estrategia de Cambio Climático y se incluyeron acciones para que las mujeres definieran las zonas prioritarias para asegurar la tenencia de tierras.
	^39^^,^^40^	En Chiapas, la percepción de las mujeres frente a la escasez de recursos les genera más preocupación. Ellas tienden a acatar las recomendaciones, pero los servicios de extensión no siempre las tienen en cuenta. Mediante procesos comerciales -como venta de productos- han adquirido oportunidades de autonomía e independencia. Las mujeres de la comunidad Shawi, en Perú, señalaron que las lluvias tempranas dificultan la quema de campos para cultivar yuca y plátano. El clima impredecible afecta la preparación de la tierra y la producción. Tradicionalmente, los hombres cazaban y las mujeres cultivaban, pero la disminución de la caza dejó a los hombres sin su rol social, mientras que los programas gubernamentales centrados en mujeres aumentan su carga laboral.
Medidas de adaptación al cambio climático y planes gubernamentales
	^29^^,^^33^^,^^38^^,^^41^^-^^43^	Los procesos agroecológicos en conjunción con los saberes ancestrales, que reconocen a las mujeres como reservorios del conocimiento, han asegurado la salud de las familias y el desarrollo personal de las mujeres. La agricultura familiar campesina en Colombia aporta a la mitigación y la adaptación al cambio climático, sustentada en el conocimiento frente al manejo de riesgos, la conservación de la agrobiodiversidad y la sostenibilidad de los recursos naturales. En Brasil, se viene adoptando un enfoque definido como *Community-driven Development*, para reducir la vulnerabilidad y, en consecuencia, aumentar su capacidad de adaptación a los efectos del cambio climático. En Perú, los pueblos indígenas están incorporando procesos de agrodiversidad que responden de manera parcial debido al consumo externo de los productos. En Cuba, se realizó la implementación de casas de postura y cultivos semiprotegidos, centros de producción de materia orgánica, minindustria, agricultura de conservación y sistemas de captación de agua de lluvia en viviendas rurales. En México, se seleccionan y usan semillas criollas, se hace asociación de cultivos, rotación y conservación de suelos, y se usa estiércol animal.
Políticas y recursos para el empoderamiento de la mujer
	^32^^-^^35^^,^^38^	Los procesos agroecológicos articulados con los saberes ancestrales y los medios de vida híbridos de campesinos e indígenas en Latinoamérica, son los recursos iniciales de adaptación al cambio climático junto con políticas públicas y las primeras cuotas de participación femenina. Los pagos por servicios ambientales, producción de plantas, reforestación y reconocimiento de la economía del cuidado representan las iniciativas de adaptación en países como Perú, Ecuador y México.
	^44^	La participación de las mujeres en los programas REDD+ sigue siendo un desafío, ya que no solo se requieren cambios políticos, sino también, de la visión de quienes los implementan. A menudo, se ignoran las desigualdades y diversidad de las mujeres, considerando el género como un tema secundario. El principal reto es que las acciones de las organizaciones civiles superen su carácter aislado y puntual.
	^45^	Hay un impacto muy positivo de incluir la perspectiva de género en los programas y de las cisternas para recoger el agua de lluvia en la vida cotidiana de las familias, porque redundan en mejores condiciones de salud, ingresos y educación, lo cual potencia también la capacidad de adaptación y contribuye a reducir la vulnerabilidad de las poblaciones más frágiles.
	^46^	Las barreras para la construcción de políticas con perspectiva de género se relacionan con la pobreza de las mujeres, lo que limita su participación política y el acceso a oportunidades. La debilidad institucional del gobierno, la brecha entre la formulación y la implementación de políticas, y el desconocimiento de lo que es «enfoque de género», se identificaron en Honduras.
	^47^	Las iniciativas políticas y de desarrollo destinadas a abordar las desigualdades de género en la agricultura y la adaptación al cambio climático, han tendido a considerar a las mujeres como grupos homogéneos cuyos derechos debían equipararse a los de los hombres rurales. A menudo, esto ha supuesto intervenciones de “talla única” que, en teoría, beneficiarían a las mujeres por igual, independientemente de otras dimensiones sociales como su estatus socioeconómico, educación, edad, raza, religión, etc.
	^48^	La mayoría de las políticas, leyes, planes y estrategias revisadas en Honduras y Guatemala, presentan enfoque de género, aunque en distintos grados. Las políticas no proponen indicadores específicos de género en sus planes de acción. Ninguna de las políticas incluyó indicadores cualitativos. Las políticas revisadas no asignaron de manera explícita recursos financieros para implementar los programas de género. Muchas de las consideraciones de género en las políticas revisadas de cambio climático, seguridad alimentaria y nutrición, permanecen generales e inespecíficas, lo que limita su potencial transformador.
	^49^	Ecuador por su geografía costera, proclive al impacto negativo de las alteraciones climáticas, requiere una acción especial de planeación de políticas de contención. Se plantea la necesidad de que los poderes públicos se impliquen de forma integral en la incorporación de políticas públicas frente al cambio climático, sin embargo hay avances en el reconocimiento de la perspectiva de género en las diferentes COP. Es ideal que los planteamientos de las politicas públicas sean territorializadas y demuestren su real impacto, con procesos de monitoreo seguimiento y evaluación constantes.

En esta revisión, se encontró que los eventos más relevantes vinculados con el cambio climático en Latinoamérica incluyen sequías, inundaciones, deslizamientos y aumentos de temperatura con potencial de provocar incendios. Estas situaciones afectan directamente la agricultura y generan vulnerabilidades económicas, sociales, ambientales y políticas. Estos eventos, cada vez más frecuentes y graves, se han asociado con pérdida de los glaciares, disminución de la disponibilidad de agua y degradación del suelo.

Su impacto en las mujeres se intensifica debido a los roles históricamente asignados, como el cuidado de los hijos y de las personas enfermas. M. Casas documenta que la escasez de agua incrementa la carga del trabajo doméstico, reduciendo el acceso a la educación y al empleo remunerado, especialmente durante los desastres y después de ellos ^33^.

Las mujeres y las niñas constituyen el grupo más afectado por el cambio climático debido a las limitaciones en el acceso a la tierra, agua, crédito, capacitación y tecnologías; esto se traduce en menores ingresos y oportunidades laborales, y en mayor exposición a la violencia de género. En las comunidades rurales e indígenas, la recolección de agua y leña -que puede tomar entre 8 y 11 horas diarias- aumenta la carga laboral y limita el acceso a la educación o las actividades remuneradas. Asimismo, se identificaron desigualdades en la distribución de los derechos de herencia y en la valoración de su aporte económico.

Entre las medidas de adaptación más relevantes, se destacan los procesos agroecológicos con enfoque de género, la integración de saberes ancestrales y la participación en los programas de mitigación climática. Sin embargo, persisten barreras estructurales en las políticas públicas y en el acceso a los recursos.

Se analizaron los estudios desde las cuatro dimensiones de la seguridad alimentaria y se resumieron en la tabla 3. En la dimensión de *disponibilidad*, las mujeres asocian el cambio climático con la reducción de alimentos y la necesidad de adoptar estrategias de diversificación productiva u orientadas a cultivos semiprotegidos para garantizar el abastecimiento de alimentos; en la dimensión de *acceso*, se identificó que la disminución de la producción obliga a destinar más ingresos a la compra de alimentos, lo que afecta especialmente a los hogares encabezados por mujeres; en la dimensión de *utilización* se observó la preferencia por los alimentos orgánicos, el autoconsumo derivado de prácticas agroecológicas y el desarrollo de productos agroprocesados con valor nutracéutico, que fortalecen la autonomía económica de las mujeres; y, finalmente, la dimensión de *estabilidad,* comprometida por fenómenos climáticos extremos, limitaciones en la conservación de alimentos y violencia de género, lo que agrava la inseguridad alimentaria en las comunidades vulnerables.

**Tabla 3:** Resultados del análisis de los artículos seleccionados según las dimensiones afectadas de la seguridad alimentaria

Dimensión	Referencias	Resultados
Disponibilidad: existencia de alimentos en cantidad suficiente y de calidad adecuada, proporcionados por medio de la producción del país o mediante importaciones, incluida la ayuda alimentaria (FAO, 2011)	^18^^,^^20^^,^^21^^,^^23^^-^^26^^,^^30^^,^^33^^,^^35^^,^^38^	El cambio climático es percibido por la mayoría de las mujeres como el causante de la afectación de la disponibilidad de alimentos. Las estrategias de adaptación asociadas con el sector agropecuario fueron clave para consolidar la disponibilidad de las comunidades rurales de Los Andes colombianos y latinoamericanos. La reducción de desperdicios de alimentos se considera una forma de adaptación al incremento de las exigencias de recursos afectados por el cambio climático. Se pueden generar medidas que contribuyan a la seguridad alimentaria, en condiciones de adaptación efectiva al cambio climático, mediante un mayor reconocimiento de las mujeres vinculadas a las áreas de cultivos semiprotegidos junto con el aumento de su productividad y la diversificación de sus cultivos. En el sector alimentario, las mujeres participan de manera significativa. La ausencia de disponibilidad de alimentos por el cambio climático incrementa el tiempo y la dificultad de las tareas domésticas.
Acceso: capacidad para adquirir los alimentos disponibles para satisfacer las necesidades propias, (incluye factores económicos y recursos físicos) (FAO, 2011).	^16^^,^^17^^,^^19^^,^^24^^,^^30^^,^^35^^,^^39^	Uno de los efectos del cambio climático es el bajo acceso económico, ya que parte de los ingresos familiares son destinados a comprar los alimentos que antes se producían en el predio. Se disminuyen los ingresos por reducción de los rendimientos. Las mujeres están más predispuestas a invertir en soluciones de adaptación al cambio climático, aunque tengan menores ingresos por actividades agrícolas y haya menos inversión en sus fincas. Las mujeres usan los recursos económicos obtenidos a partir de prácticas y tecnologías de agricultura sostenible adaptada al clima, para la compra de comida. La intensificación sostenible en la producción de alimentos y un mayor acceso a la tenencia de tierra por parte de las mujeres, permitirán el acceso físico y económico adecuado. Usualmente, las mujeres obtienen algunos alimentos para sus familias a partir de productos forestales no maderables. Debido a un panorama económico difícil, la migración es el medio que tienen las mujeres para lograr el acceso a los alimentos. Las madres solteras tienen menor disponibilidad por la falta de recursos económicos.
Utilización: uso biológico de los alimentos, que vincula el estado nutricional y de salud (FAO, 2011)	^21^^,^^22^^,^^24^^,^^32^	La mejora de la alimentación familiar fue el mayor beneficio de la producción agroecológica, ya que los productos se destinan primero para el autoconsumo, lo que a su vez los lleva a comprometerse con los programas que gestionan los recursos. Las mujeres reconocen que, debido al cambio climático, se usan muchos productos químicos en alimentos y sienten la necesidad de buscar productos más orgánicos. La carencia de medios de conservación de los alimentos (cadena de frío) obstaculiza la calidad microbiológica de los mismos. Existe una mayor capacitación y fomento productivo a mujeres campesinas para el desarrollo de productos agroprocesados con valor nutracéutico o alimentos multifuncionales (mermeladas, jugos, aceites esenciales, hierbas medicinales, entre otros) que utilicen prácticas sostenibles, a fin de fomentar la autonomía económica y, con ello, la autoestima y el sentimiento de bienestar consigo misma, su familia y la comunidad. Estudios en diversos países en vía de desarrollo muestran cómo las mujeres experimentan inseguridad alimentaria y deficiencias nutricionales porque la comida es redistribuida de manera preferente hacia los otros miembros de la familia.
Estabilidad: garantía de disponibilidad, acceso y uso adecuado de los alimentos por parte de la población. Esta dimensión es transversal a las otras tres dimensiones.	^17^^,^^20^^-^^23^^,^^32^^-^^36^^,^^38^^,^^39^^,^^45^^,^^46^	Las mujeres acceden menos a créditos para desarrollar sus actividades agrícolas, pero cuando lo hacen, son más propensas a invertirlos en hacer frente a los riesgos del cambio climático que los hombres. La aplicación de prácticas de agricultura climáticamente inteligentes (*CSA* en inglés) -por parte de las mujeres- se asocia positivamente con el nivel educativo y el grado de cultivo, pero estas asociaciones están condicionadas por la diversificación de los medios de subsistencia y el tipo de tenencia de la tierra. La violencia de genero conlleva a la escasez de recursos ambientales, lo que genera que las mujeres también presenten inseguridad alimentaria. Las sequias producto del cambio climático afectan la salud y el bienestar de las mujeres, al obligarlas a realizar búsquedas de recursos más lejanas y prolongadas. Las razones de no usar la información climática recibida tienen diferencias de género. Tanto hombres como mujeres perciben mayor producción agrícola y mayores ingresos derivados de la implementación de las prácticas de agricultura sostenible adaptada al clima. Cuando las mujeres se empoderan financieramente, son más proclives a invertir en alimentos.

En la revisión de Alpino *et al*. ^43^, se destaca que los efectos negativos del cambio climático sobre la seguridad alimentaria y nutricional se han documentado principalmente en los países en desarrollo, donde se agravan las condiciones de desnutrición y las deficiencias nutricionales, y paralelamente, el sobrepeso y la obesidad. Por el contrario, en los países desarrollados, la atención se centra en la calidad e inocuidad microbiológica de los alimentos. Los impactos más relevantes identificados se relacionan con el acceso, la producción, la calidad nutricional y la variabilidad de los precios de los alimentos, que afectan principalmente a las poblaciones en situación de desigualdad social.

## Discusión

### El cambio climático no es neutro respecto al género: evidencias de las brechas existentes

El objetivo del presente estudio fue presentar la evidencia disponible sobre la relación entre el cambio climático y la seguridad alimentaria en sus cuatro dimensiones, bajo una perspectiva de género en Latinoamérica. Se consideraron las categorías de análisis: vulnerabilidad, roles de género, medidas de adaptación y recursos o políticas para el empoderamiento de la mujer.

Los resultados frente a estas cuatro categorías sugieren que, en Latinoamérica, el cambio climático impacta negativamente y de manera diferenciada según el género, intensificando las desigualdades preexistentes en la región ^43^. El análisis de las relaciones de género y su intersección con variables como etnia, edad y clase, han sido esenciales para entender fenómenos como la vulnerabilidad social, la resiliencia, las estrategias de adaptación, la percepción del riesgo y el papel de los saberes locales frente al cambio climático ^50^.

Los resultados de la revisión de Alpino *et al.*^43^ indican que, para que las intervenciones agrícolas y de adaptación al cambio climático sean eficaces y respondan a las necesidades de las mujeres rurales, es imprescindible reconocer su heterogeneidad y comprender cómo el género interactúa con otras dimensiones sociales, configurando distintos grados de vulnerabilidad ^51^.

El superar estas desigualdades requiere un enfoque ético y socioambiental adaptado a las realidades locales, que reconozca que las mujeres asumen una mayor carga en el uso y el mantenimiento de los recursos naturales, lo que incrementa su exposición a los impactos climáticos; además, enfrentan menor acceso a la educación, la información y los servicios básicos ^52^.

En relación con las políticas y los recursos frente al cambio climático, varios artículos incluidos ^14^^,^^24^^,^^25^^,^^46^ evidencian brechas persistentes en la integración de la perspectiva de género en las estrategias de adaptación e investigación agrícola. Como señalan Quisumbing *et al.*^53^, es necesario incluir indicadores claros para medir los avances en la reducción de las brechas de género, así como en el acceso a los recursos y a la toma de decisiones, lo que se refleja en la capacidad de respuesta ante las crisis climáticas.

Al analizar la desigualdad en la región, se observa que es más acentuada en las áreas rurales, donde las tasas de pobreza alcanzan el 45,7 % y la pobreza extrema el 21,7 %, superando por dos y tres veces las cifras urbanas; además, el 76 % de la población ocupada en el campo se encuentra en informalidad laboral. Las zonas rurales registran ingresos bajos, menor acceso a servicios y baja escolaridad ^54^. En este contexto, las brechas de género reproducen vulnerabilidades sociales, y dificultan el éxito de políticas públicas frente al cambio climático y a las secuelas de la pandemia de COVID-19 ^55^.

En el Programa de las Naciones Unidas para las Mujeres, se afirma que tanto las mujeres como las niñas sufren de manera desproporcionada los efectos del cambio climático, lo que amplifica las desigualdades, y amenaza sus medios de vida, salud y seguridad ^56^. Estos riesgos son especialmente graves para mujeres indígenas, afrodescendientes y adultas mayores, personas LGBTIQ+, mujeres con discapacidad, migrantes y quienes residen en zonas rurales o propensas a desastres o conflictos. Durante las sequías y lluvias irregulares, las agricultoras aumentan su carga laboral para obtener ingresos y recursos, mientras que las niñas abandonan sus estudios para colaborar en las tareas de supervivencia. Entre los ejemplos más críticos, está la mayor afectación de las mujeres indígenas debido a prácticas discriminatorias históricas, sumadas a factores como la ubicación geográfica, el analfabetismo y las barreras lingüísticas, que dificultan el acceso a la información sobre mitigación y prevención de desastres, lo cual puede desencadenar desplazamientos forzados hacia las zonas urbanas ^57^. Por lo anterior, los enfoques interseccionales son relevantes al estudiar el impacto del cambio climático en la seguridad alimentaria.

Aguilar ^15^ señala que las mujeres tienen menor acceso a los recursos productivos y tecnológicos, y que la violencia de género opera como barrera para la conservación con enfoque de derechos y el desarrollo sostenible ^58^. Según la Comisión Económica para América Latina y el Caribe (CEPAL) ^59^, la recopilación de datos específicos sobre violencia hacia las mujeres en situaciones de desplazamiento o migración ambiental sigue siendo prioritaria.

Las personas migrantes pueden encontrarse en situaciones de vulnerabilidad por condiciones asociadas con su movilidad, estatus legal y calidad de vida que, en algunos casos, funcionan como estrategia de adaptación frente a las presiones locales ^60^. Sin embargo, esta migración suele derivarse de la pobreza estructural, con migración masculina en busca de empleo, lo que deja a las mujeres con doble carga laboral -agrícola y doméstica- o con migración femenina y juvenil que reconfigura la estructura familiar ^60^^,^^61^.

Estas dinámicas, sumadas a las pérdidas agrícolas, reducen la disponibilidad de alimentos y obligan a reorganizar el hogar, razón por la cual las mujeres asumen la responsabilidad de la tierra, el ganado y el cuidado familiar. Uno de los hallazgos más constantes es que las actividades de cuidado y las domésticas no remuneradas recaen predominantemente sobre las mujeres, lo cual limita su acceso a los ingresos y reduce su participación en los espacios comunitarios.

Respecto a las cuatro dimensiones de la seguridad alimentaria, en la presente revisión se encontró que la disponibilidad de alimentos se ve comprometida por efectos directos e indirectos del cambio climático en los sistemas alimentarios, con variaciones regionales determinadas por la vulnerabilidad del territorio y la población ^62^.

En los estudios incluidos realizados en países latinoamericanos, como Perú, México, Bolivia, Colombia, Honduras, Costa Rica, Nicaragua, Ecuador, Guatemala, Chile y Brasil, las sequías, las inundaciones, los incrementos de temperatura y los deslizamientos fueron los eventos más frecuentes, todos asociados con disminuciones en la producción y con una consecuente falta de abastecimiento.

Los hallazgos de la presente revisión coinciden con los de Alpino y Silva ^62^, ya que los impactos directos del cambio climático incluyen pérdidas en plantaciones y ganadería, disminución de productividad agrícola y aumento en los precios de alimentos básicos. Sin embargo, esta revisión aporta evidencia adicional sobre el impacto diferenciado en las mujeres rurales, quienes en varios estudios reportaron pérdida de ingresos y mayor carga de trabajo no remunerado, lo que dificulta su participación en las actividades productivas.

La dimensión de estabilidad también resulta afectada, ya que el clima influye en la producción y en la oferta y la demanda de alimentos, lo cual genera variaciones de precios que impactan principalmente a los países con mayores índices de pobreza ^62^. En las regiones costeras, por ejemplo, las mujeres desempeñan un papel clave en la pesca artesanal y en su procesamiento; sin embargo, su baja representación en la planificación costera limita la inclusión de sus intereses en las políticas públicas, lo cual afecta el consumo de alimentos de mayor valor nutricional ^27^.

En la presente revisión se confirma que la variabilidad de los precios afecta con mayor fuerza a las poblaciones vulnerables, dado que destinan una mayor proporción de sus ingresos a la compra de alimentos y tienen menor capacidad para modificar sus patrones de consumo. Esto las obliga a optar por productos de menor calidad nutricional, comprometiendo la seguridad alimentaria ^62^. La situación se agrava para las mujeres en condición de pobreza, ya que la dependencia económica puede limitar su acceso a mecanismos de protección frente a la violencia de género. Como señalan Aguilar ^58^ y UN Women ^56^, el acceso limitado a los recursos y alimentos aumenta la exposición a situaciones de abuso, lo cual dificulta la denuncia y perpetúa los ciclos de vulnerabilidad que impactan su seguridad personal y alimentaria.

### De la vulnerabilidad a las medidas de adaptación al cambio climático con enfoque de género

En la presente revisión se identificaron diversas medidas de adaptación y respuesta implementadas frente a la situación crítica que enfrentan las mujeres en el contexto del cambio climático. Por ejemplo, en Cuba ^41^, se desarrollaron talleres participativos municipales con enfoque de género, liderados por grupos técnicos, que permitieron reconocer acciones para cerrar las brechas identificadas en los diagnósticos sobre la adaptación climática ^56^^,^^62^. En Ecuador, la Fundación para la Protección Ecológica (FUNDECOL) impulsó la conservación de los manglares mediante la participación de las mujeres, fortaleciendo su rol en la gestión comunitaria de los recursos naturales ^49^. En Honduras, un ejemplo significativo de adaptación fue la gestión comunitaria de una expendedora de agua potable a bajo costo que, además, generó empleo parcial para las mujeres solteras con hijos y los excedentes se destinaron a proyectos barriales ^56^^,^^57^. En Brasil, el enfoque *community-driven development* busca reducir la pobreza rural, como estrategia para fortalecer las medidas de adaptación climática ^57^.

De los 36 artículos analizados, solo en ocho se mencionaron explícitamente los recursos diferenciados con que cuentan los hombres y las mujeres para afrontar el cambio climático, como el acceso a internet, los programas educativos, los talleres especializados y las redes de apoyo comunitarias. Un caso destacado en México fue la creación de la “Troika+ de mujeres líderes sobre género y cambio climático”, una red que articula mujeres y hombres comprometidos con promover medidas de adaptación con perspectiva de género ^56^.

Entre las estrategias reportadas, se contemplan: aumentar la participación femenina en los cargos para la toma de decisiones ambientales, evaluar las políticas y los programas ambientales desde una perspectiva de género y aplicar la legislación que prohíbe la violencia contra la mujer, incluyendo las directrices y los planes nacionales para la igualdad ^56^^,^^59^. También, se prioriza el acceso de las mujeres a la tierra, los recursos productivos, la educación y el trabajo digno e igualitario, así como su inclusión en los proyectos productivos y ambientales gubernamentales, reconociendo estos como herramientas para mejorar su resiliencia ^56^^,^^59^.

En síntesis, los hallazgos de esta revisión confirman que los impactos del cambio climático -sequías, inundaciones, deslizamientos y variabilidad de los patrones de lluvia- afectan de manera desproporcionada a las mujeres rurales e indígenas, quienes desempeñan un papel central en la producción y la gestión de alimentos. Esta vulnerabilidad se intensifica por las desigualdades estructurales, como el acceso limitado a los recursos, la educación y la participación en la gobernanza climática. No obstante, los estudios también evidencian su rol como agentes clave en la mitigación y la adaptación al cambio climático, aunque frecuentemente invisibilizado. Por lo tanto, resulta prioritario adoptar enfoques interseccionales que consideren el género, la etnia, la clase y la edad, así como fortalecer las políticas públicas que garanticen la equidad, optimicen los sistemas alimentarios locales y promuevan la participación de las mujeres en la gobernanza climática ^56^^,^^59^^,^^57^^,^^63^.

En Latinoamérica serán necesarias iniciativas que promuevan la participación efectiva en la toma de decisiones, la gestión territorial y el acceso equitativo a las tecnologías y el financiamiento climático que protegan no solo a las mujeres sino a las familias y sus entornos cotidianos, favoreciendo el derecho humano a la alimentación y la justicia climática. ^63^. En este sentido, la COP30 de noviembre del 2025, celebrada en Brasil, ha adoptado el nuevo plan de acción de género 2026-2034 que se convierte en el instrumento operativo para promover la igualdad de género en las políticas, planes y acciones climáticas ^64^. Asimismo, muchas de las demandas planteadas por las mujeres se reflejan en la Declaración Ministerial de Lima, en la cual los gobiernos de Latinoamérica y el Caribe reconocen la necesidad de cerrar las brechas de género y reconocer las contribuciones de las mujeres a la acción ambiental y climática ^64^.
